# Autologous Osteochondral Grafting From an Ipsilateral Ankle Osteophyte for Chronic and Large Osteochondral Lesion of the Talus

**DOI:** 10.1155/cro/7925783

**Published:** 2026-03-25

**Authors:** Yasunari Ikuta, Tomoyuki Nakasa, Saori Ishibashi, Satoru Sakurai, Dan Moriwaki, Taro Chujo, Nobuo Adachi

**Affiliations:** ^1^ Department of Orthopaedic Surgery, Hiroshima University Hospital, Hiroshima, Japan, hiroshima-u.ac.jp; ^2^ Department of Artificial Joints and Biomaterials, Graduate School of Biomedical and Health Sciences, Hiroshima University, Hiroshima, Japan, hiroshima-u.ac.jp

## Abstract

Large osteochondral lesions of the talus (OLTs) in the chronic phase remain challenging to manage due to degeneration of the osteochondral fragment and underlying subchondral bone defects, which often make conventional fixation techniques difficult to perform. Although various surgical approaches—including autologous osteochondral transplantation, chondrocyte implantation, and structural allografting—have been employed, each carries limitations such as the requirement for an additional donor‐site harvest, the need for staged procedures, or limited graft availability. We report the case of a 19‐year‐old male with a chronic lateral OLT unresponsive to conservative treatment. Imaging revealed a large, multifragmented, and detached osteochondral fragment unsuitable for fixation. Surgical treatment was performed under ultrasound‐guided peripheral nerve block and involved osteochondral reconstruction using an autologous graft harvested from a large osteophyte at the ipsilateral anterior tibial plafond. The graft was carefully shaped to match the articular contour of the defect and fixed with three bioabsorbable pins. The postoperative course was uneventful, and the patient returned to preinjury levels of sports activity. At 19‐month follow‐up, he remained symptom‐free, with excellent scores on clinical outcome scales and the self‐administered foot evaluation questionnaire. Imaging confirmed successful graft integration and joint congruity, with no signs of graft resorption or implant‐related complications. This case highlights the potential utility of osteophyte‐derived autologous grafting as a single‐stage, minimally invasive alternative for large OLTs, particularly when the native osteochondral fragment is nonviable and an appropriately sized osteophyte is available. This technique may reduce overall surgical invasiveness by avoiding the need for graft harvesting from distant donor sites, offering a promising treatment option in selected cases.

## 1. Introduction

Osteochondral lesions of the talus (OLTs) can cause significant pain and functional impairment and often require surgical intervention for symptomatic lesions [1]. Treatment strategies vary depending on the lesion size, location, and subchondral bone conditions [2, 3]. For small lesions (< 10 mm diameter, < 100 mm^2^ area), bone marrow stimulation techniques such as microfracture are commonly employed [4, 5]. Fixation of the osteochondral fragment is recommended to preserve joint congruity for OLTs with maintained articular surfaces [6]. In contrast, large lesions (> 10 mm diameter or > 100 mm^2^ area) typically necessitate more advanced reconstructive techniques [7]. Osteochondral autografting and allografting are appropriate for restoring large defects, offering superior anatomical contours and mechanical strength [1]. Autologous matrix‐induced chondrogenesis has demonstrated efficacy in reducing pain, improving ankle function, and facilitating return to sports [8, 9]. Other emerging therapies include particulated juvenile cartilage allograft transplantation, which promotes hyaline‐like cartilage regeneration [10], and cell‐based therapies such as autologous chondrocyte implantation (ACI) [1].

Despite these advances, chronic OLTs with large lesions remain particularly challenging due to degenerative osteochondral fragments and poor underlying subchondral bone quality. In such cases, reconstructive procedures that restore both the articular surface and subchondral bone are often required. Talar osteoperiostic grafting from the iliac crest (TOPIC), which involves transplantation of a corticoperiosteal autograft harvested from the iliac crest to reconstruct the osteochondral defect, has shown excellent clinical outcomes and is considered a promising treatment option for large OLTs in both male and female patients [11]. Although donor‐site morbidity following iliac crest harvesting for TOPIC has been reported to be limited [11], these reconstructive techniques typically necessitate additional surgical access for graft harvesting from distant donor sites. This requirement increases the overall procedural complexity, including the potential for scarring or discomfort at the donor‐site [12]. Therefore, identifying alternative autologous graft sources with osteochondral potential that can be harvested within the primary operative field is desirable to minimize surgical invasiveness.

Recently, Kawabata et al. proposed the use of osteophyte cartilage as an alternative source for minced cartilage implantation. Their study demonstrated that osteophyte cartilage exhibits characteristics similar to native articular cartilage, including comparable expression of cartilage‐specific genes, chondrocyte proliferation capacity, and glycosaminoglycan synthesis. These findings suggest that osteophytes could serve as a viable autologous source for chondrocyte‐based cartilage repair [13].

Based on this concept, we explored the feasibility of harvesting osteophyte cartilage from the ipsilateral ankle to reconstruct a large OLT. In this report, we present a case of chronic OLT involving a large, detached, and multifragmented osteochondral fragment with degenerative cartilage. The lesion was successfully treated using an osteochondral graft harvested from a large osteophyte of the ipsilateral ankle, resulting in excellent clinical outcomes.

## 2. Case Presentation

A 19‐year‐old man initially sprained his left ankle during elementary school and subsequently experienced multiple ankle sprains. Although he had recurrent sprains since childhood, he had no persistent ankle pain until 3 years prior to presentation, when he sustained a supination ankle sprain while playing baseball, which marked the onset of persistent left ankle pain. Despite conservative management at the clinic of origin, his symptoms progressively worsened, particularly during sports activities, and he was referred to our outpatient clinic.

On physical examination, localized tenderness was noted at the anterolateral (AL) aspect of the ankle without swelling. A reproducible palpable click was noted at the AL aspect of the ankle during circumduction, which may indicate a symptomatic intra‐articular condition, such as an unstable osteochondral lesion. Range of motion was limited to 15° dorsiflexion and 40° plantarflexion. Manual stress testing revealed ankle instability on both varus stress and anterior drawer maneuvers. The score on the Japanese Society for Surgery of the Foot (JSSF) ankle‐hindfoot scale [14, 15] was 69 points.

Weight‐bearing plain radiographs demonstrated an osteochondral lesion at the lateral talar dome, accompanied by sclerosis of the subchondral bone and detached osteochondral fragments (Figure 1a, b). Osteophyte formation was observed at the anterior tibial plafond and dorsal aspect of the talar neck. Computed tomography (CT) revealed separated osteochondral fragments (Figures 1c, 1d, and 1e), with the underlying bone defect measuring 22 mm in length and 13 mm in width, yielding an estimated area of 225.9 mm^2^ [16]. Magnetic resonance imaging (MRI) on T2‐weighted images showed a thinned remnant of the anterior talofibular ligament (ATFL). Based on these findings, a diagnosis of a chronic OLT accompanied by lateral ankle instability was made.

Figure 1Preoperative weight‐bearing plain radiographs (a, b) and computed tomographic images (c–e). Detached osteochondral fragments (red circle) and a large osteophyte from the anterolateral tibial plafond (red arrow) are shown.

**Figure:**
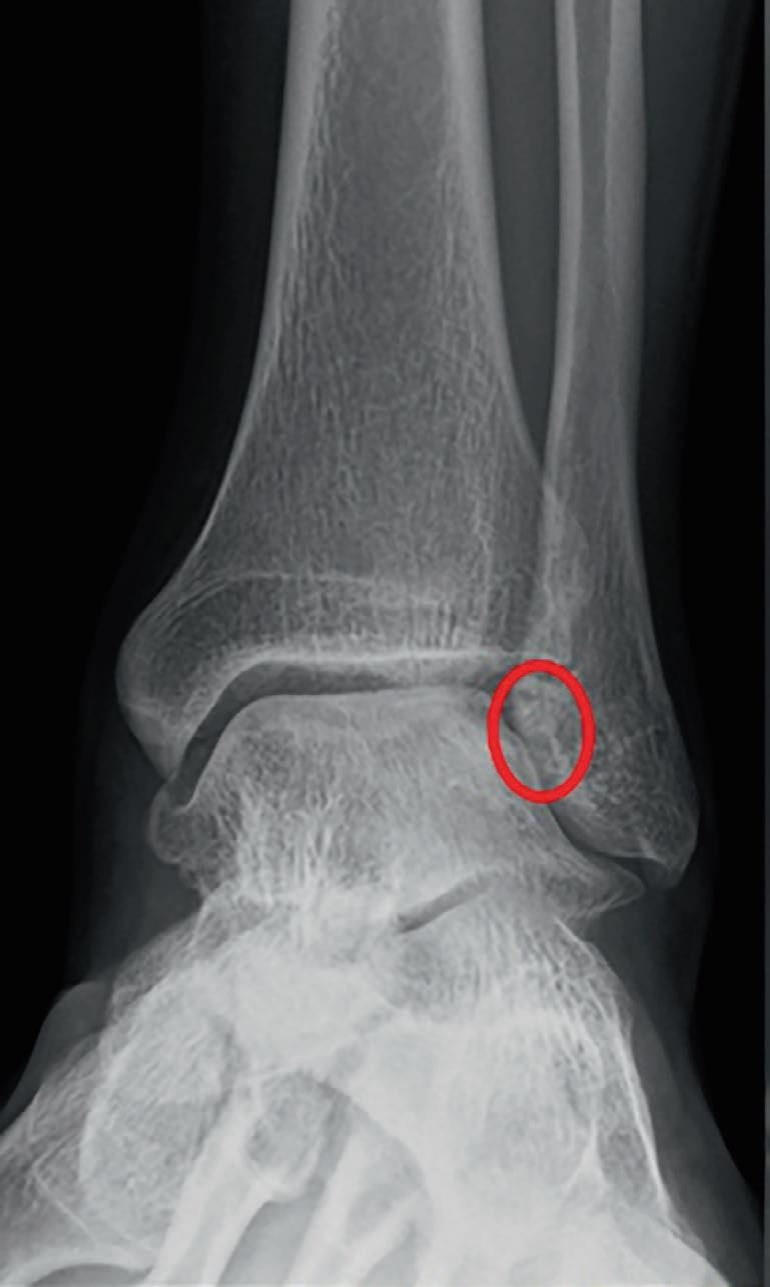
(a)

**Figure:**
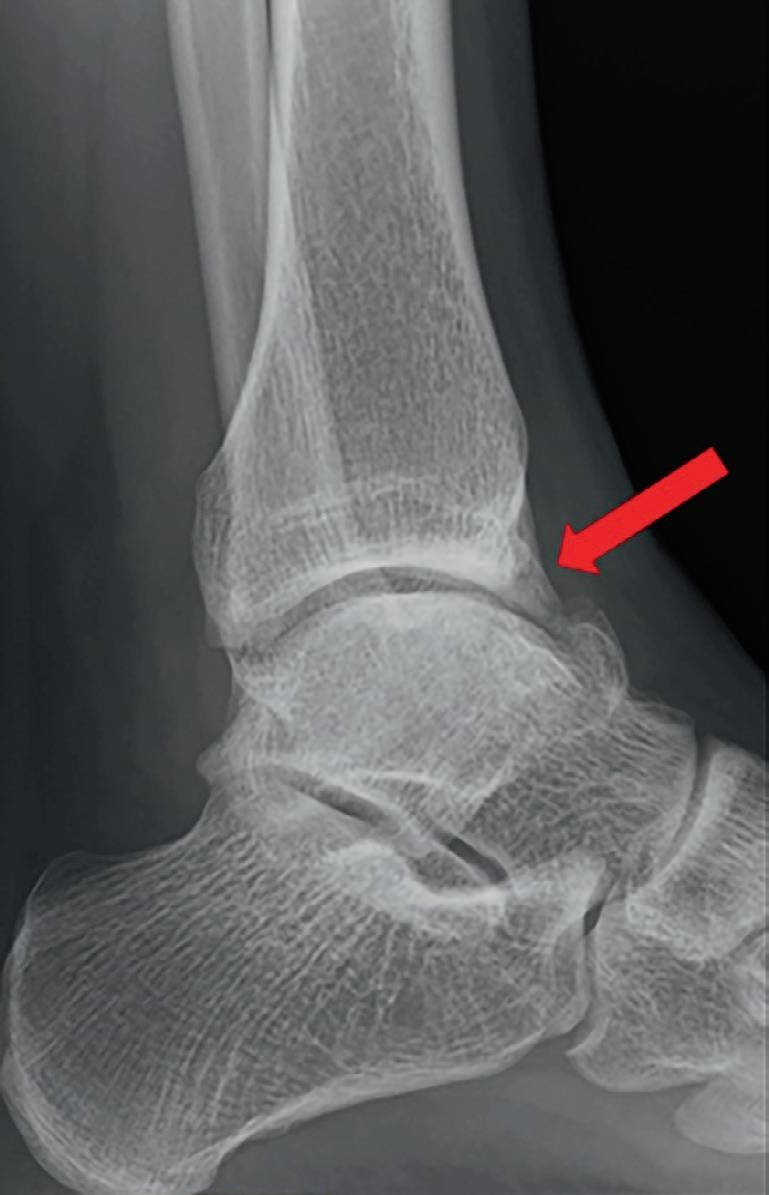
(b)

**Figure:**
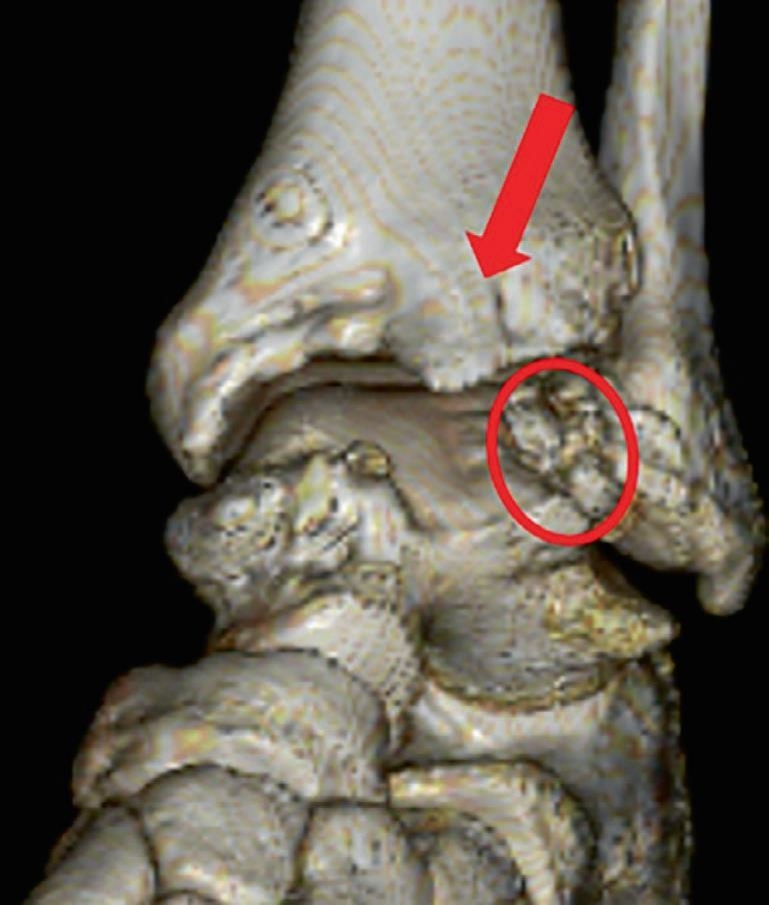
(c)

**Figure:**
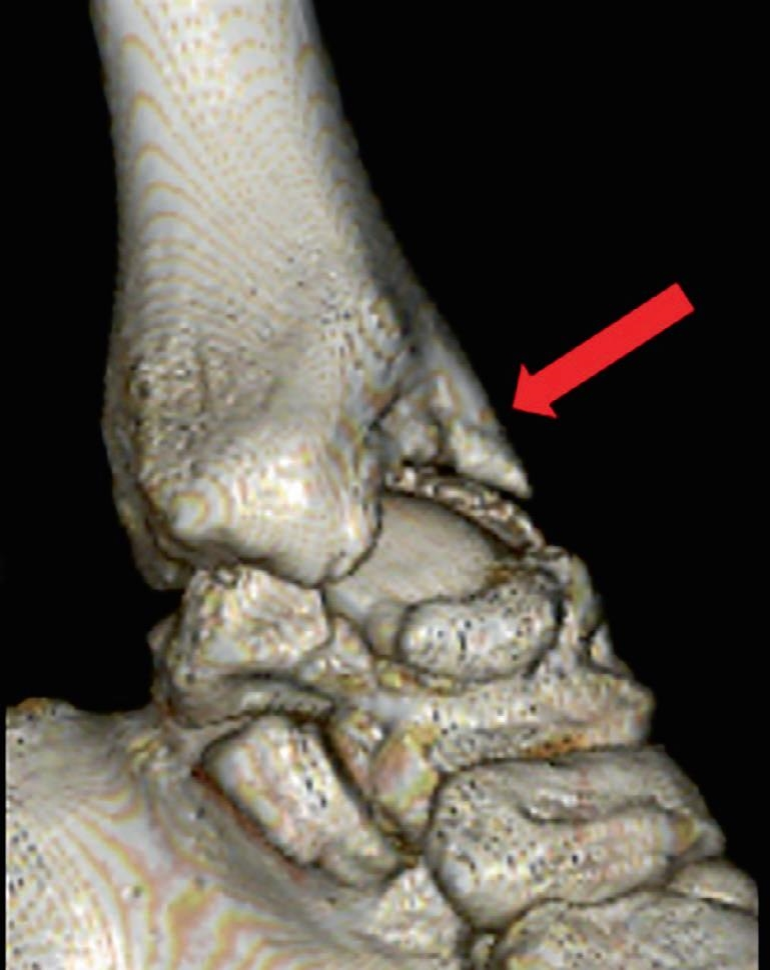
(d)

**Figure:**
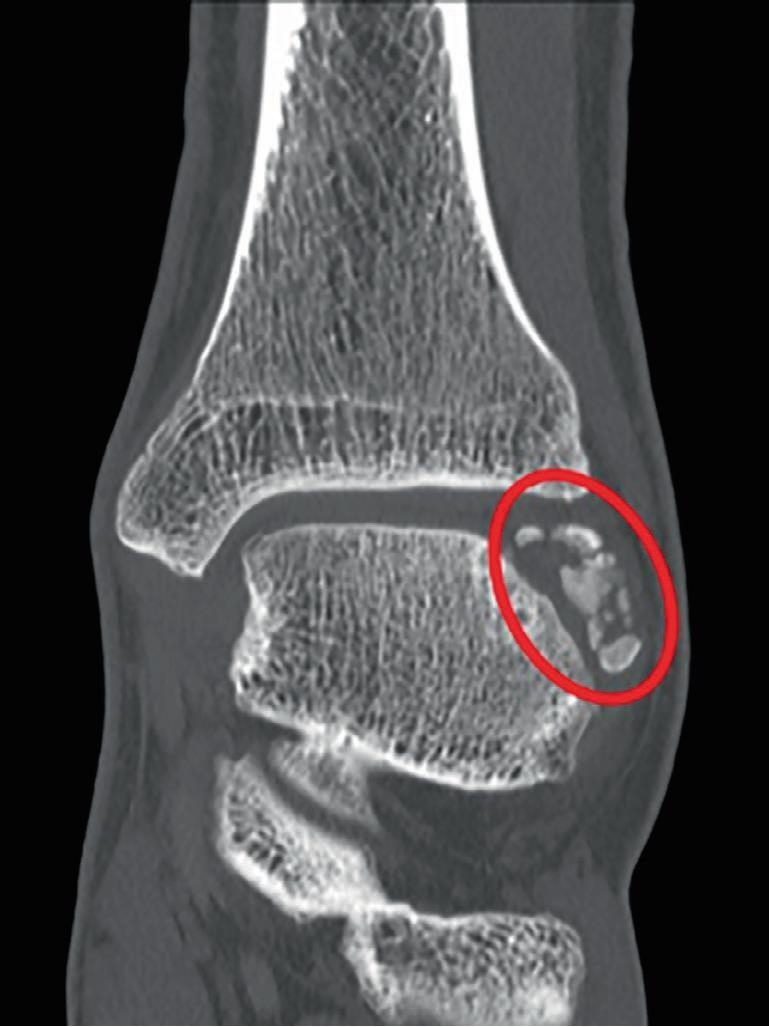
(e)

### 2.1. Surgical Technique

Surgical treatment was performed under ultrasound‐guided peripheral nerve blocks, specifically targeting the sciatic and saphenous nerves, without sedation. Conventional AL and anteromedial portals were placed under joint distraction using an ankle distractor (Smith & Nephew, Memphis, Tennessee, United States), and a 2.7‐mm, 30° oblique arthroscope was used for intra‐articular evaluation. Arthroscopy revealed detached and unstable osteochondral fragments with degenerative cartilage in the AL zone (Figure 2). The AL portal was longitudinally extended to create a mini‐open approach, allowing direct access to the osteochondral lesion for osteophyte resection and graft fixation. The chondral fragment was degenerated and separated multifragmentary (Figure 3a, b). Thus, it would be challenging to fix each osteochondral fragment. Chondral fragments were extracted from the joint.

Figure 2Arthroscopic view through anteromedial portal (a, b). Detached and multifragmented osteochondral fragments with degenerative cartilage in the anterolateral zone.

**Figure:**
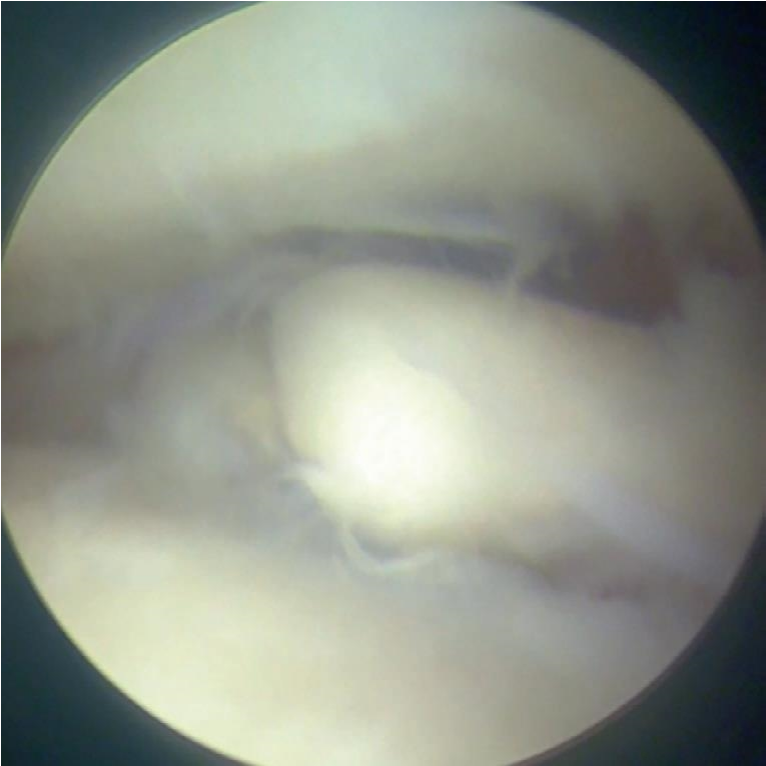
(a)

**Figure:**
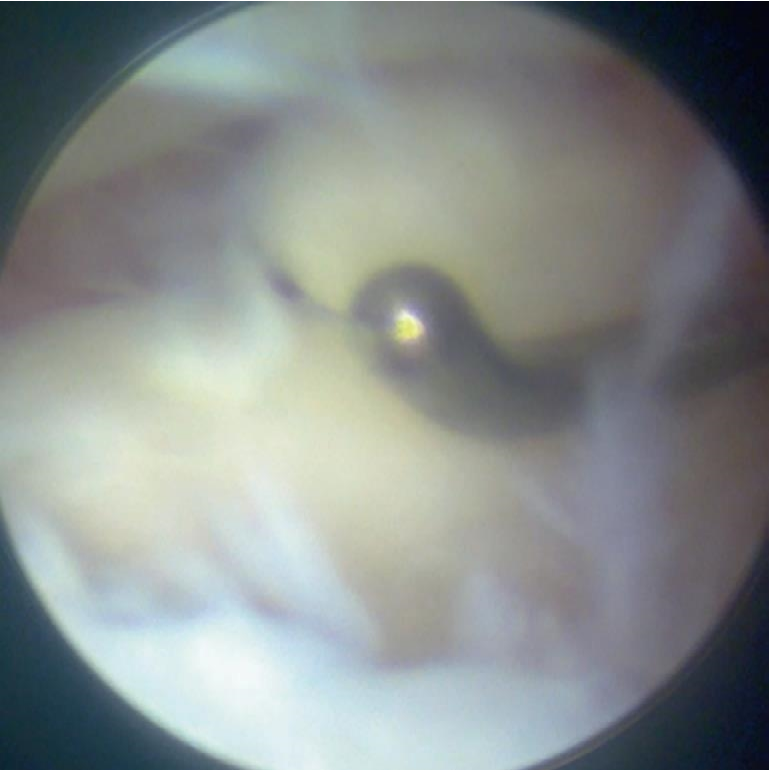
(b)

Figure 3Intraoperative images from extended anterolateral portal (a–d). Large osteophyte of the anterolateral tibial plafond (black arrow) and detached osteochondral fragments (arrowhead) (a). Osteochondral lesion after osteophyte resection (b). Osteochondral defect after curettage of bone sclerosis and multiple drillings (c). Fixation of the osteophyte‐derived osteochondral fragment using three 1.5‐mm poly‐L‐lactic acid‐hydroxyapatite composite pins (d).

**Figure:**
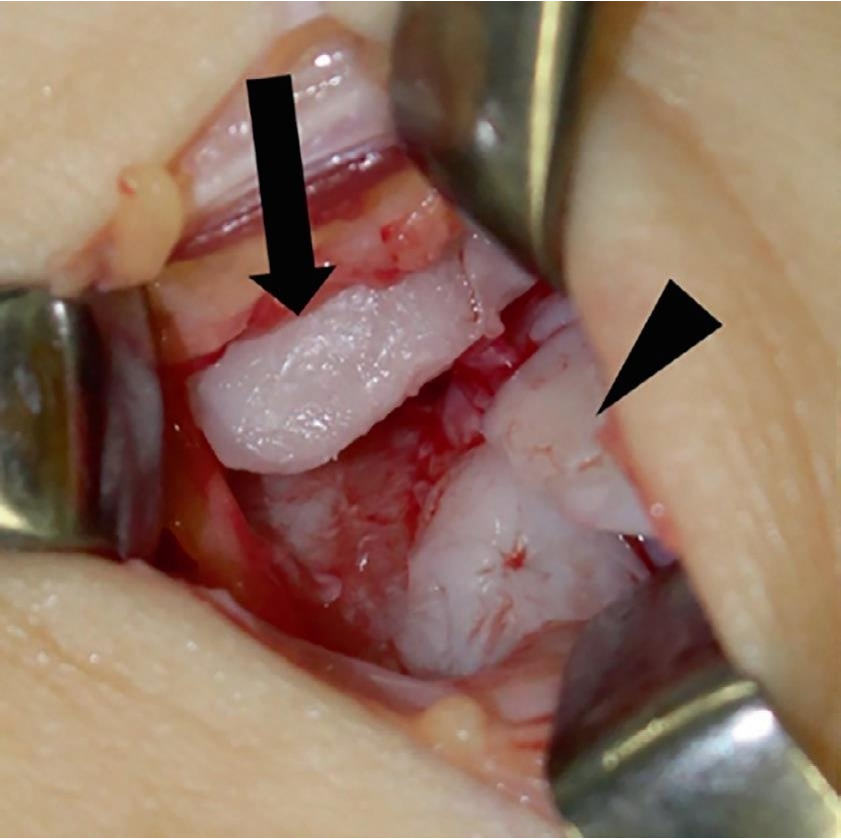
(a)

**Figure:**
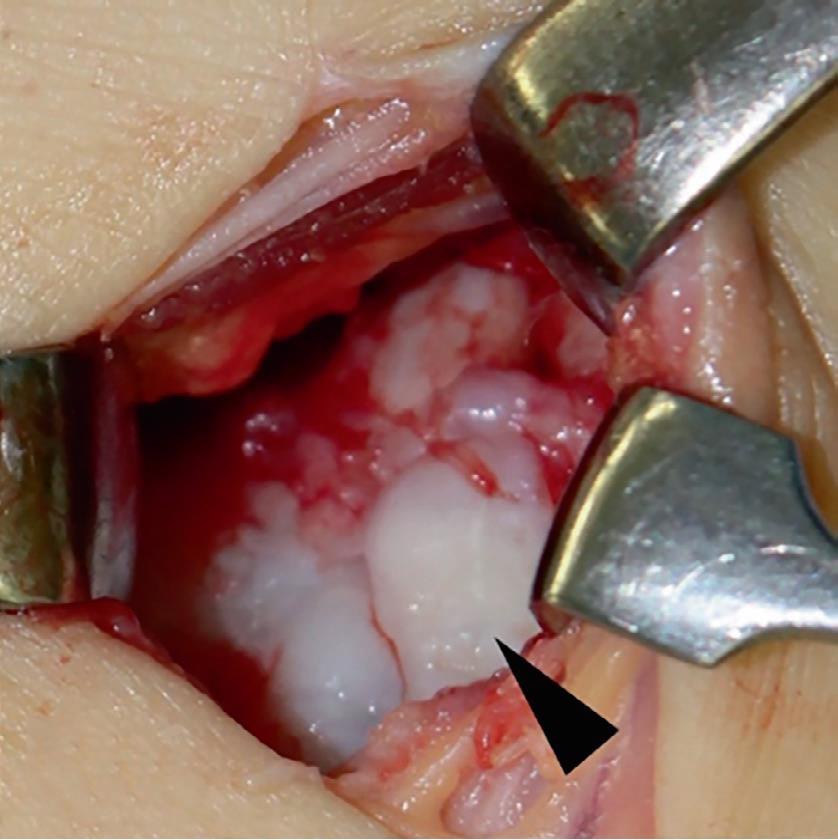
(b)

**Figure:**
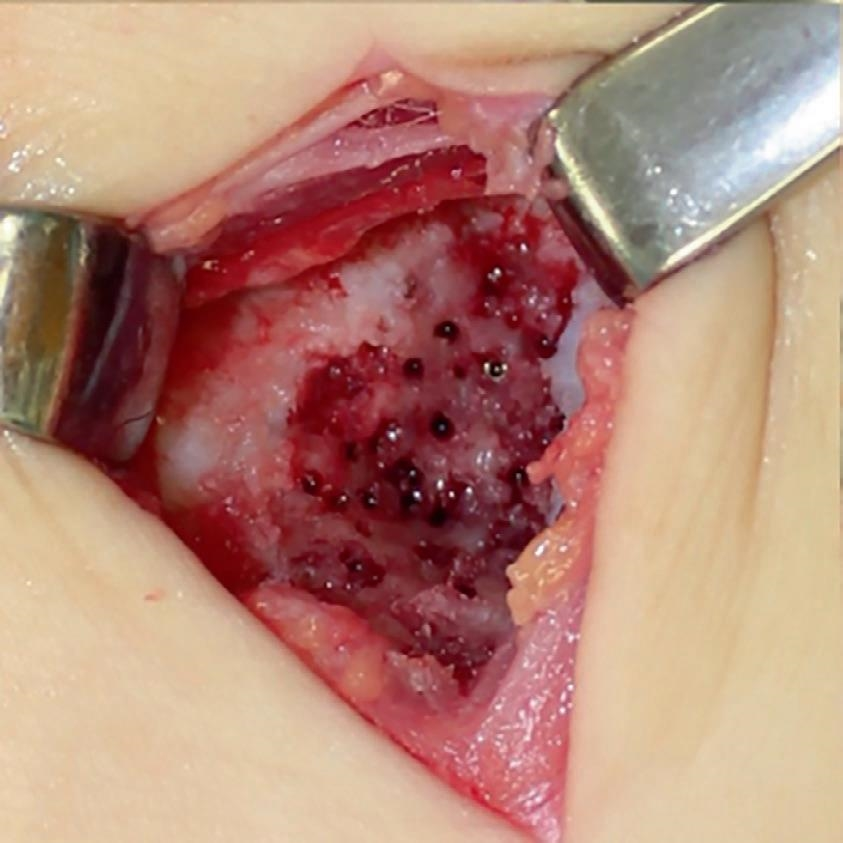
(c)

**Figure:**
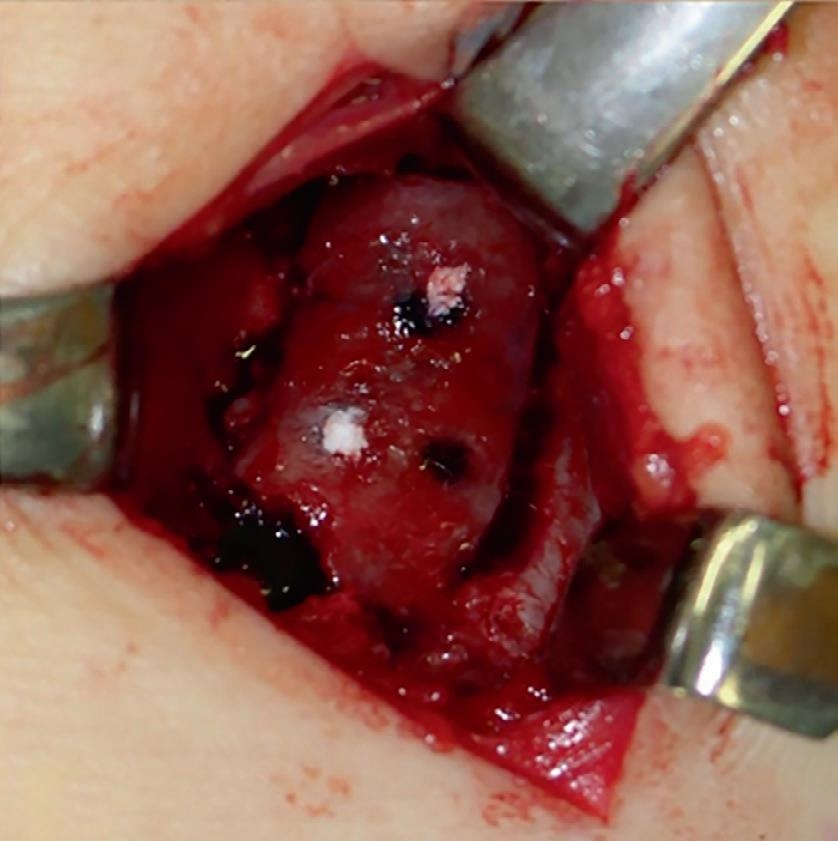
(d)

**Figure 4:**
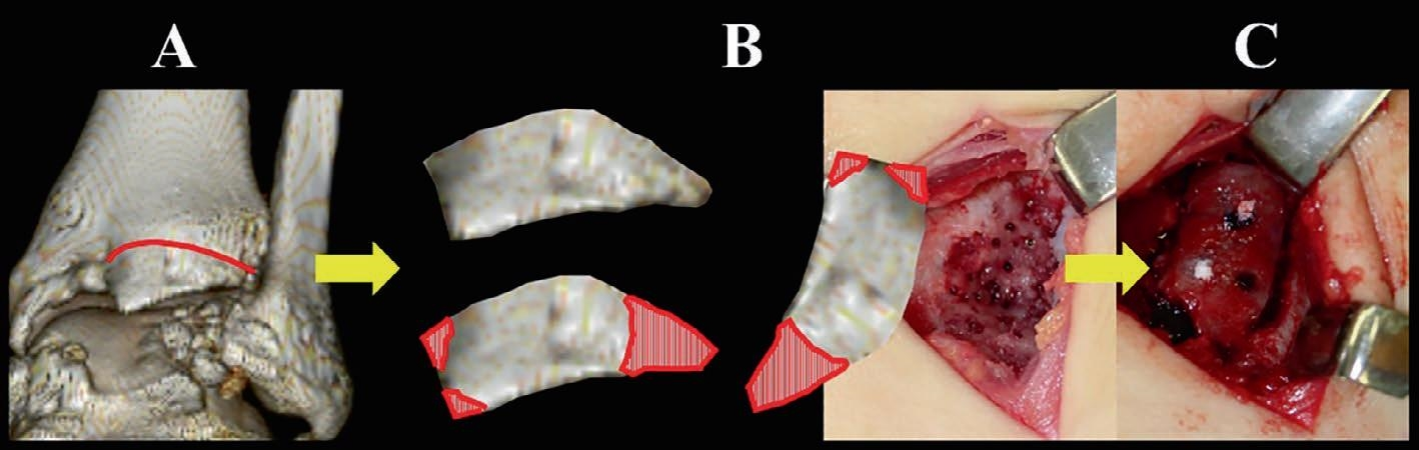
Schematic illustration of the autologous osteochondral grafting using a large ipsilateral ankle osteophyte (A–C). Resection of the osteophyte from the anterolateral tibial plafond (red line) (A). The large osteophyte was shaped to match the size of the osteochondral defect and curvature of the surrounding articular cartilage (red vertical stripes); during this preparation, the protruding anterior portion was resected (B). The graft was then rotated approximately 90° clockwise to cover the osteochondral defect with the osteophyte‐derived osteochondral fragment (C).

Osteophytes at the anterior tibial plafond, talar neck, and medial and lateral malleoli were removed using a flat chisel and a Luer rongeur. A large osteophyte from the AL tibial plafond was harvested and shaped to fit the size of the osteochondral defect and curvature of the surrounding articular cartilage (Figure 4). Cancellous bone chips were prepared from resected osteophytes. After removing covered fibrous tissue from the subchondral bone, multiple drillings were performed using a 1.0‐mm Kirschner wire. Areas of severe sclerosis, particularly in the anterior part of the defect, were curetted until viable cancellous bone was exposed (Figure 3c). Following debridement, autologous cancellous bone chips were impacted into the defect to reconstruct a stable subchondral bed. The osteophyte‐derived graft was then carefully oriented to match the surrounding articular curvature and placed flush with the adjacent native cartilage to ensure optimal joint congruity. Temporary fixation was achieved with 1.0‐ and 1.5‐mm Kirschner wires, and definitive fixation was performed using three 1.5‐mm poly‐L‐lactic acid‐hydroxyapatite composite pins (SuperFIXSORB30; TEIJIN MEDICAL TECHNOLOGIES) inserted into the anteromedial, AL, and posterior aspects of the graft to a depth of 20 mm (Figure 3d).

Subsequently, a suture tape (Ultratape, Smith & Nephew) was passed through the proximal ATFL remnant and capsule using a racking hitch technique and secured at three sites. A drill hole was created at the anatomical footprint of the superior fascicle of the ATFL on the fibula. A knotless anchor (Bioraptor, Smith & Nephew) with the suture tape was inserted into the fibula at the neutral position of the ankle. Varus stress with a force of approximately 50 N was manually provided to the ankle joint, and the absence of instability was confirmed under fluoroscopy.

### 2.2. Postoperative Course

Postoperative rehabilitation was supervised by a physical therapist. Physical therapy was initiated on postoperative Day 1, consisting of toe motion and conditioning exercises of the uninvolved joints and lower limb, whereas the ankle was immobilized in a posterior plaster splint. After 3 weeks of immobilization, ankle range‐of‐motion exercises were started, followed by progressive strengthening and proprioceptive/balance training. Partial and full weight bearing were permitted at 5 and 8 weeks, respectively. Jogging and return to sports were allowed at 4 and 6 months postoperatively, respectively.

At the 19‐month follow‐up, he had returned to presymptomatic level of sports participation without pain or swelling. The JSSF ankle‐hindfoot score had improved to 100. The Self‐Administered Foot Evaluation Questionnaire [17] demonstrated subscale scores of 100 for pain and pain‐related symptoms, social function, shoe‐related issues, general health and well‐being, 95.5 for physical function and daily living, and 96.7 for sports activity. The 7‐month radiograph demonstrated radiographic consolidation of the grafted area, and the 1‐year radiograph showed maintained joint congruity of the lateral talar dome (Figure 5a, b). MRI obtained at 1 year demonstrated findings consistent with graft incorporation and revealed no signs of osteolysis around the bioabsorbable pins (Figure 5f, g). CT scans obtained at 19 months confirmed complete osseous union of the graft and congruent restoration of the lateral talar dome (Figures 5c, 5d, and 5e).

Figure 5Postoperative weight‐bearing plain radiographs (a, b), computed tomographic (c–e) and magnetic resonance images (f, g). Bone union of the graft and congruent restoration of the lateral talar dome are observed (red arrow). No evidences of osteolysis around the bioabsorbable pins (f, g).

**Figure:**
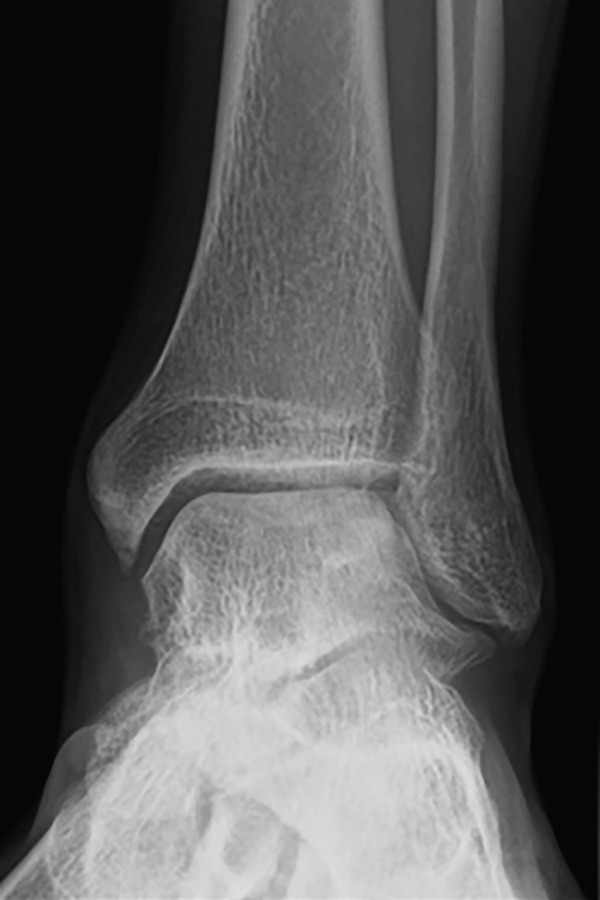
(a)

**Figure:**
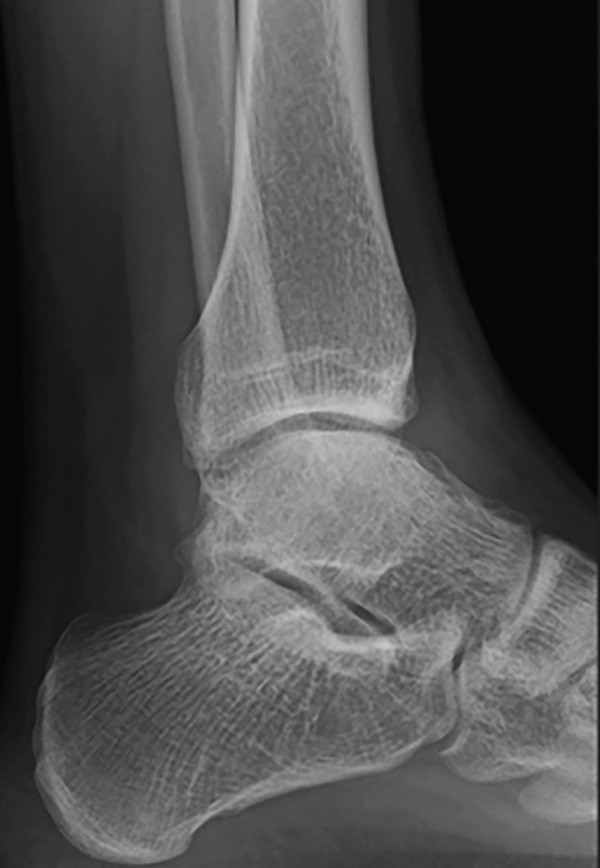
(b)

**Figure:**
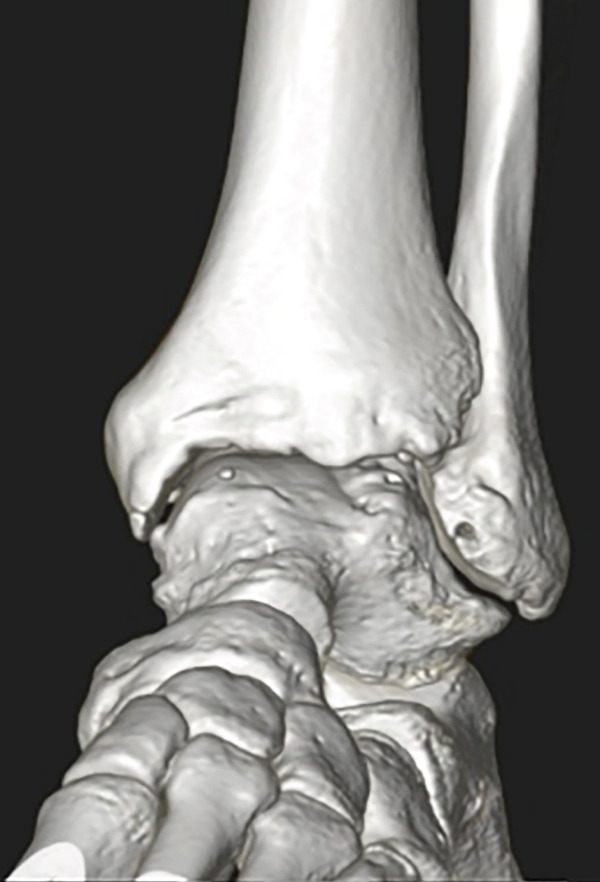
(c)

**Figure:**
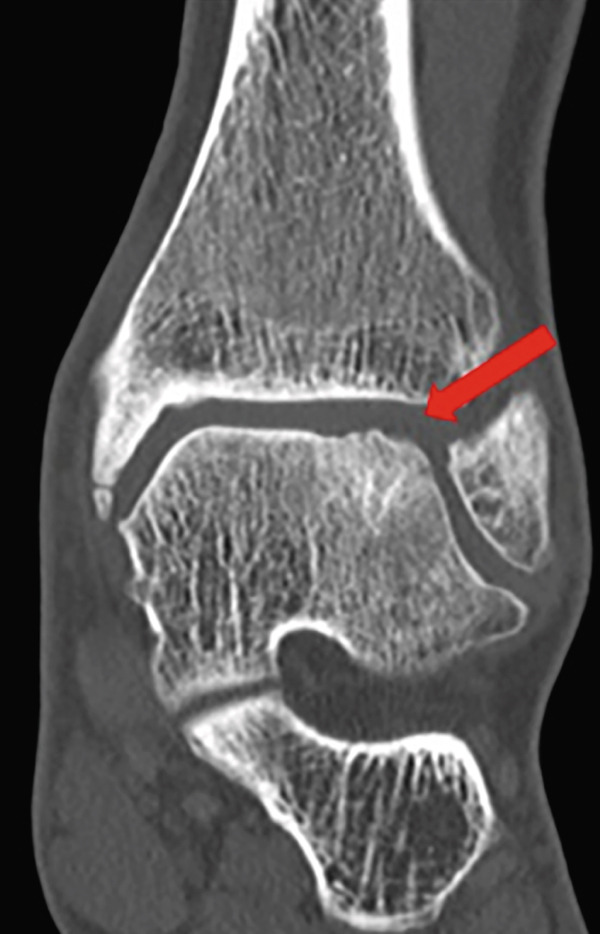
(d)

**Figure:**
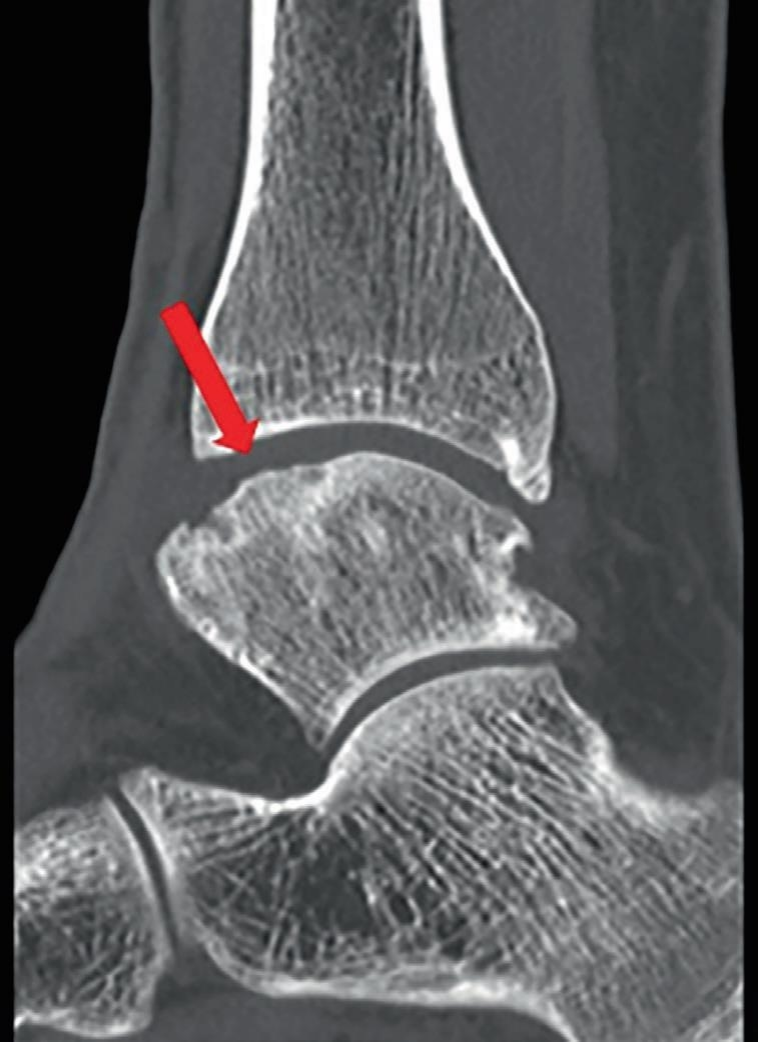
(e)

**Figure:**
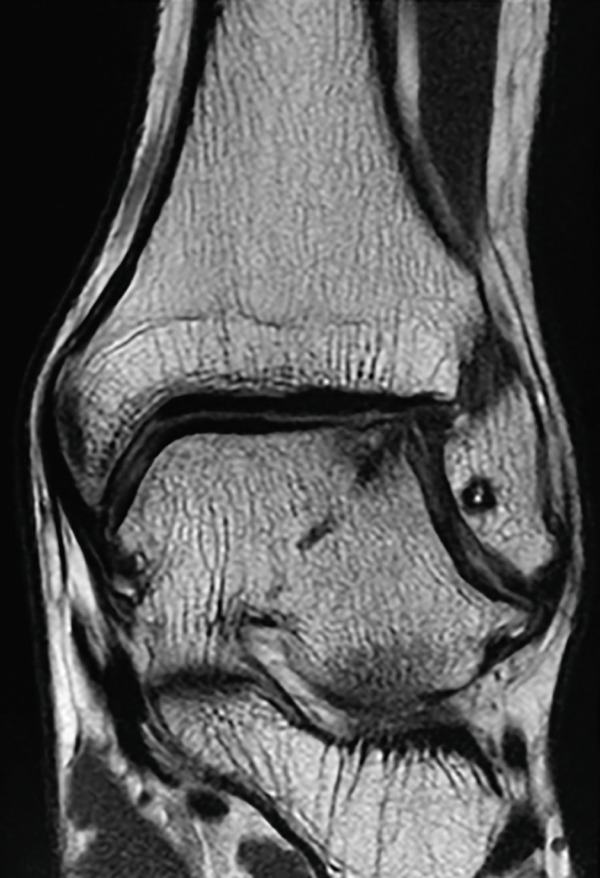
(f)

**Figure:**
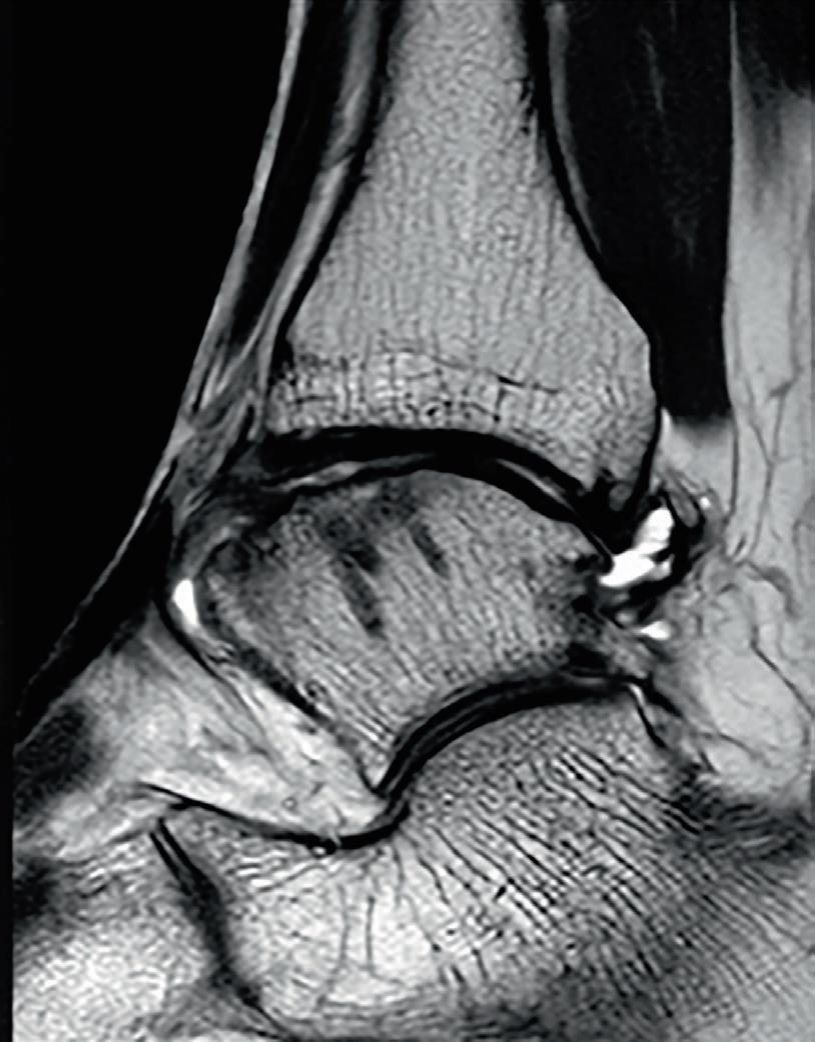
(g)

## 3. Discussion

The treatment of large OLTs in the chronic phase remains challenging due to the degeneration and fragmentation of osteochondral fragments, as well as subchondral bone defects. Autologous osteochondral grafting using osteophytes may represent a viable option for chronic and large OLTs, particularly when a sufficiently large osteophyte from the ipsilateral ankle is available for graft preparation. This approach yielded excellent short‐term postoperative outcomes in the present case. In contrast to conventional osteochondral reconstruction techniques that require graft harvesting from distant donor sites, the use of locally available osteophytes reduces surgical invasiveness by avoiding an additional donor‐site procedure while achieving acceptable joint congruity.

Several surgical options have been proposed for managing chronic large OLTs, including autologous osteochondral transplantation (AOT), ACI, and structural osteochondral allograft. However, each technique has concerns in this situation. The AOT procedure, particularly when multiple plugs are required for large lesions, can lead to donor‐site morbidity and persistent pain [18]. Although ACI has been shown to provide good clinical results, it necessitates a two‐step procedure. Structural allografting offers immediate restoration of joint congruity; however, it often carries the risk of graft failure and may require revision surgery [19, 20].

Osteophytes have been shown to possess high bone mineralization potential and exhibit increased osteoblast activity. Moreover, osteophytes secrete various growth factors, including transforming growth factor‐*β*1, bone morphogenetic protein‐2, and insulin‐like growth factor‐1 [21]. Kawabata et al. have reported the potential of osteophyte‐derived cartilage as a source for minced cartilage implantation. Their comparative analysis revealed that chondrocytes from osteophyte cartilage exhibited comparable migration, proliferation, and matrix production capacities to those from normal articular cartilage. Gene expression levels of COL2A1, ACAN, and SOX9 were similar between the two sources, and both demonstrated robust glycosaminoglycan production. These findings support the use of osteophyte cartilage as an alternative autologous source for cartilage repair [13].

In the present case, a large osteophyte from the anterior tibial plafond was harvested and used for osteochondral reconstruction. The graft was shaped to match the curvature and dimensions of the defect, with the convex surface aligned to the surrounding normal articular cartilage. This technique allowed for a single‐stage, minimally invasive procedure without the need for additional skin incisions or graft harvesting from distant donor sites, thereby avoiding an additional donor‐site procedure.

This report has several limitations. First, as this is a single‐case experience with a relatively short follow‐up period of 19 months, the generalizability and long‐term durability of the technique remain to be established. Second, the procedure is only feasible in selected patients who present with a sufficiently large osteophyte with an adequate osteochondral surface in the ipsilateral ankle, which may limit its broader clinical applicability. Further studies involving larger cohorts and longer follow‐up periods are warranted to confirm the long‐term clinical outcomes of this approach.

Given the successful return to preinjury sports activities with excellent clinical function and maintained radiographic joint congruity without signs of graft resorption at 19‐month follow‐up, autologous osteochondral grafting using ipsilateral ankle osteophytes may represent a promising alternative treatment for chronic large OLTs with degenerative fragments. This technique is particularly advantageous when native osteochondral fragments are unsuitable for fixation and an osteophyte of adequate size and curvature is available within the ipsilateral ankle joint.

## Funding

No funding was received for this manuscript.

## Consent

Written informed consent was obtained from the patient and the patient′s parents for publication of this case report and any accompanying images.

## Conflicts of Interest

The authors declare no conflicts of interest.

## Data Availability

The data that support the findings of this study are available from the corresponding author upon reasonable request.

## References

[1] Wang C. C. , Yang K. C. , and Chen I. H. , Current Treatment Concepts for Osteochondral Lesions of the Talus, Tzu Chi Medical Journal. (2021) 33, no. 3, 243–249, 10.4103/tcmj.tcmj_106_20. 34386361 PMC8323653

[2] Steele J. R. , Dekker T. J. , Federer A. E. , Liles J. L. , Adams S. B. , and Easley M. E. , Osteochondral Lesions of the Talus, Foot & Ankle Orthopaedics. (2018) 3, 10.1177/2473011418779559. PMC10408332 37566685

[3] Anastasio A. T. , Bagheri K. , Peairs E. M. , Grant C. , and Adams S. B. , Juvenile Osteochondral Lesions of the Talus: Current Concepts Review and an Update on the Literature, Children. (2023) 10, no. 5, 10.3390/children10050884. PMC10217026 37238431

[4] Ramponi L. , Yasui Y. , Murawski C. D. , Ferkel R. D. , DiGiovanni C. W. , Kerkhoffs G. M. , Calder J. D. , Takao M. , Vannini F. , Choi W. J. , and Lee J. W. , Lesion Size Is a Predictor of Clinical Outcomes After Bone Marrow Stimulation for Osteochondral Lesions of the Talus: A Systematic Review, American Journal of Sports Medicine. (2017) 45, no. 7, 1698–1705, 10.1177/0363546516668292, 2-s2.0-85020179746. 27852595

[5] Hannon C. P. , Bayer S. , Murawski C. D. , Canata G. L. , Clanton TO , Haverkamp D. , Lee J. W. , O’Malley M. J. , Yinghui H. , and Stone J. W. , Debridement, Curettage, and Bone Marrow Stimulation: Proceedings of the International Consensus Meeting on Cartilage Repair of the Ankle, Foot Ankle International. (2018) 39, 16S–22S, 10.1177/1071100718779392, 2-s2.0-85049908749. 30215307

[6] Nakasa T. , Ikuta Y. , Haraguchi N. , Park C. H. , Weber C. D. , Rikken Q. G. , Dahmen J. , Stufkens S. A. , Kerkhoffs G. M. , and Takao M. , An Evidence-Based Update on Fixation Procedures for Acute and Chronic Osteochondral Lesions of the Talus, Cartilage. (2024) 17, 88–100, 10.1177/19476035241280072. 39311645 PMC11556605

[7] Butler J. J. , Robert G. , Dahmen J. , Lin C. C. , Robin J. X. , Samsonov A. P. , Kerkhoffs G. M. , and Kennedy J. G. , Outcomes Following Autologous Osteochondral Transplantation for Osteochondral Lesions of the Talus at 10-Year Follow-Up: A Retrospective Review, Cartilage. (2024) 17, no. 1, 10.1177/19476035241293268. PMC11556656 39469788

[8] Weigelt L. , Hartmann R. , Pfirrmann C. , Espinosa N. , and Wirth S. H. , Autologous Matrix-Induced Chondrogenesis for Osteochondral Lesions of the Talus: A Clinical and Radiological 2- to 8-Year Follow-up Study, American Journal of Sports Medicine. (2019) 47, no. 7, 1679–1686, 10.1177/0363546519841574, 2-s2.0-85067011446. 31084491

[9] Jiang N. , Li H. , Wang J. , Shen L. , and Zeng X. , The Efficacy of Autologous Matrix-Induced Chondrogenesis (AMIC) for Osteochondral Lesions of the Talus in the Mid-Long Term: A Systematic Review and Meta-Analysis, Journal of Orthopaedic Surgery and Research. (2024) 19, no. 1, 10.1186/s13018-024-04864-z. PMC11194938 38915104

[10] Adams S. B. , Demetracopoulos C. A. , Parekh S. G. , Easley M. E. , and Robbins J. , Arthroscopic Particulated Juvenile Cartilage Allograft Transplantation for the Treatment of Osteochondral Lesions of the Talus, Arthroscopy Techniques. (2014) 3, no. 4, e533–e537, 10.1016/j.eats.2014.06.004, 2-s2.0-85027920588. 25264516 PMC4175163

[11] Dahmen J. , Gianakos A. L. , Hollander J. J. , Rikken Q. G. H. , Stufkens S. A. S. , and Kerkhoffs G. , Sex-Specific Analysis in Patients Undergoing Talar OsteoPeriostic Grafting From the Iliac Crest (TOPIC) for Large Osteochondral Lesions of the Talus, Knee Surgery, Sports Traumatology, Arthroscopy. (2024) 32, no. 10, 2679–2687, 10.1002/ksa.12257. 38796727

[12] Dimitriou R. , Mataliotakis G. I. , Angoules A. G. , Kanakaris N. K. , and Giannoudis P. V. , Complications Following Autologous Bone Graft Harvesting From the Iliac Crest and Using the RIA: A Systematic Review, Injury. (2011) 42, no. Suppl 2, S3–15, 10.1016/j.injury.2011.06.015, 2-s2.0-80052838150. 21704997

[13] Kawabata S. , Nakasa T. , Nekomoto A. , Yimiti D. , Miyaki S. , and Adachi N. , Osteophyte Cartilage as a Potential Source for Minced Cartilage Implantation: A Novel Approach for Articular Cartilage Repair in Osteoarthritis, International Journal of Molecular Sciences. (2024) 25, no. 10, 10.3390/ijms25105563. PMC11122408 38791601

[14] Niki H. , Aoki H. , Inokuchi S. , Ozeki S. , Kinoshita M. , Kura H. , Tanaka Y. , Noguchi M. , Nomura S. , Hatori M. , and Tatsunami S. , Development and Reliability of a Standard Rating System for Outcome Measurement of Foot and Ankle Disorders I: Development of Standard Rating System, Journal of Orthopaedic Science. (2005) 10, no. 5, 457–465, 10.1007/s00776-005-0936-2, 2-s2.0-26244449518. 16193356 PMC2797841

[15] Niki H. , Aoki H. , Inokuchi S. , Ozeki S. , Kinoshita M. , Kura H. , Tanaka Y. , Noguchi M. , Nomura S. , Hatori M. , and Tatsunami S. , Development and Reliability of a Standard Rating System for Outcome Measurement of Foot and Ankle Disorders II: Interclinician and Intraclinician Reliability and Validity of the Newly Established Standard Rating Scales and Japanese Orthopaedic Association Rating Scale, Journal of Orthopaedic Science. (2005) 10, no. 5, 466–474, 10.1007/s00776-005-0937-1, 2-s2.0-26244431740. 16193357 PMC2797857

[16] Choi W. J. , Park K. K. , Kim B. S. , and Lee J. W. , Osteochondral Lesion of the Talus: Is There a Critical Defect Size for Poor Outcome?, American journal of Sports Medicine. (2009) 37, no. 10, 1974–1980, 10.1177/0363546509335765, 2-s2.0-70649102000. 19654429

[17] Niki H. , Tatsunami S. , Haraguchi N. , Aoki T. , Okuda R. , Suda Y. , Takao M. , and Tanaka Y. , Validity and Reliability of a Self-Administered Foot Evaluation Questionnaire (SAFE-Q), Journal of Orthopaedic Science. (2013) 18, no. 2, 298–320, 10.1007/s00776-012-0337-2, 2-s2.0-84879684696. 23299996 PMC3607735

[18] Nguyen A. , Ramasamy A. , Walsh M. , McMenemy L. , and Calder J. D. F. , Autologous Osteochondral Transplantation for Large Osteochondral Lesions of the Talus Is a Viable Option in an Athletic Population, American Journal of Sports Medicine. (2019) 47, no. 14, 3429–3435, 10.1177/0363546519881420. 31671274

[19] Adams S. B. , Dekker T. J. , Schiff A. P. , Gross C. P. , Nunley J. A. , and Easley M. E. , Prospective Evaluation of Structural Allograft Transplantation for Osteochondral Lesions of the Talar Shoulder, Foot Ankle International. (2018) 39, 28–34, 10.1177/1071100717732342, 2-s2.0-85040018443. 28971693

[20] Gaul F. , Barr C. R. , McCauley J. C. , Copp S. N. , and Bugbee W. D. , Outcomes of Salvage Arthrodesis and Arthroplasty for Failed Osteochondral Allograft Transplantation of the Ankle, Foot Ankle International. (2019) 40, 537–544, 10.1177/1071100718824082, 2-s2.0-85060926111. 30698469

[21] Ishihara K. , Okazaki K. , Akiyama T. , Akasaki Y. , and Nakashima Y. , Characterisation of Osteophytes as an Autologous Bone Graft Source: An Experimental Study in Vivo and in Vitro, Bone Joint Research. (2017) 6, 73–81, 10.1302/2046-3758.62.BJR-2016-0199.R1, 2-s2.0-85018392670. 28148490 PMC5331175

